# Coherent Superpositions of Photon Creation Operations and Their Application to Multimode States of Light

**DOI:** 10.3390/e23080999

**Published:** 2021-07-31

**Authors:** Nicola Biagi, Saverio Francesconi, Alessandro Zavatta, Marco Bellini

**Affiliations:** 1 Istituto Nazionale di Ottica (CNR-INO), L.go E. Fermi 6, 50125 Firenze, Italy; nicola.biagi@ino.cnr.it (N.B.); saverio.francesconi@ino.cnr.it (S.F.); alessandro.zavatta@ino.cnr.it (A.Z.); 2 LENS and Department of Physics & Astronomy, University of Firenze, 50019 Sesto Fiorentino, Italy

**Keywords:** quantum optics, single-photon addition, entanglement

## Abstract

We present a concise review of recent experimental results concerning the conditional implementation of coherent superpositions of single-photon additions onto distinct field modes. Such a basic operation is seen to give rise to a wealth of interesting and useful effects, from the generation of a tunable degree of entanglement to the birth of peculiar correlations in the photon numbers and the quadratures of multimode, multiphoton, states of light. The experimental investigation of these properties will have an impact both on fundamental studies concerning, for example, the quantumness and entanglement of macroscopic states, and for possible applications in the realm of quantum-enhanced technologies.

## 1. Introduction

The operation of increasing by exactly one unit the number of excitations in a bosonic quantum field is one of the most fundamental and well known of quantum physics. Mathematically, it is expressed by the action of a creation operator ${\hat{a}}^{†}$ acting on a well-defined single mode of the field. In recent years, this fundamental quantum tool has moved from the textbooks of quantum mechanics to the laboratory, where it was first implemented and applied to an optical field in 2004 [1,2]. The simple application of the creation operator to an arbitrary state of light turns it into a nonclassical state [3], thanks to the removal of the vacuum component in its density matrix expressed in the basis of Fock states [4]. After its first experimental realization, photon addition has become an extraordinary new tool for manipulating light at the deepest level and, together with photon subtraction [5,6,7,8], it has allowed reaching an impressive degree of control in the engineering of the quantum state of light. Besides having been used for the verification of fundamental quantum rules [9,10,11], photon addition and subtraction, together with sequences and superpositions of these two operations, have allowed researchers to implement many new purely-quantum protocols, such as noiseless amplification [12], state orthogonalization and arbitrary continuous-variable (CV) qubit generation [13], emulation of Kerr nonlinearities at the single-photon level [14], etc.

Just as the operation of adding a photon to a light field in a well-defined single mode has had such a dramatic impact on extending the possibilities of photonic quantum technologies, in the same way, we expect that the ability to coherently add a single photon to two or more different light modes by the superposition $\sum_{m}c_{m}{\hat{a}}_{m}^{†}$ (where the subscript *m* indexes the different modes and the $c_{m}$ are complex coefficients) may open new avenues in multimode quantum state manipulation and control.

One common feature of the application of a superposition of photon addition operations to multimode states of light is that entanglement is invariably generated at the output, independently on the input. This is in striking contrast with the effect of coherent photon subtractions, which are known to enhance and distill the entanglement already present among the modes, but not to generate entanglement from scratch [15,16]. In much the same way, and differently from photon addition, single-mode photon subtraction by the operator $\hat{a}$ may de-Gaussify and enhance the nonclassicality of a state [5,6], but it cannot produce nonclassical behavior if it was not already present in the input state.

The coherent addition of a single photon to multiple modes is thus the simplest way to generate entanglement among modes containing arbitrary, even classical and uncorrelated, states of light. It has already proved itself a powerful tool for studying fundamental quantum physics, and we expect it to become an invaluable resource also for the development of future quantum-enhanced technologies.

In the following, we present the scheme that we developed to experimentally implement such a coherent superposition of photon additions on different field modes in a heralded, non-deterministic way. It is based on stimulated parametric down-conversion (PDC) in nonlinear crystals and the indistinguishability among the different possible origins of the photons heralding the addition process. We briefly review the underlying mechanism and some of the applications of the scheme to different kinds of input states, showing the wide variety of interesting and useful effects it may generate.

## 2. Materials and Methods

### 2.1. Heralded Single-Photon Addition by PDC

The core of these experiments is the process of parametric down-conversion in a $\chi^{\left(2\right)}$-nonlinear crystal (β-barium borate BBO, type I). The crystal is pumped by a 150 mW average power UV beam obtained by frequency doubling (SHG) the 1.5 ps pulses at 785 nm coming from a mode-locked Ti:sapphire laser operating at a repetition rate of about 82 MHz.

When no fields, apart from the pump, are injected in the the crystal, spontaneous PDC of a single pump photon results in the emission of signal and idler photon pairs whose characteristics are highly quantum correlated because of energy and momentum conservation. The detection of one photon of the pair in a particular spatial and spectrotemporal mode thus precisely localizes the twin photon in space and time, if the pump characteristics are well known. We normally operate in a frequency-degenerate configuration, where the signal and idler photons are emitted at the same wavelength of the laser source. The BBO crystal is slightly tilted from the collinear configuration so that the idler and signal photons are emitted in symmetric directions along a cone with an aperture angle of ∼$3^{\circ }$.

In order to remotely prepare a pure single photon state in the signal mode [17,18], idler photons undergo narrow spatial and frequency filtering (F) by passing through etalon interference filters and coupling into a single-spatial-mode fiber before detection by an on–off avalanche photodetector (D). In fact, the remotely prepared signal state only approaches a pure state if the filter transmission function is much narrower than the momentum and spectral widths of the pump [19,20,21,22].

If an arbitrary light state is injected in the BBO crystal along the signal mode instead of vacuum, stimulated emission takes place. In this case, a click from the idler detector heralds the generation of the single-photon-added version of the input state [1,2].

It is worth noting that the rate of stimulated emission, in the low parametric gain regime, grows as $1+\bar{n}$ compared to the spontaneous rate, where $\bar{n}$ is the mean photon number of the light state in the input signal mode. Therefore, by just measuring the ratio of stimulated to spontaneous herald detection events, one may obtain an absolute intensity calibration of the state in the signal mode [23,24]. This approach has been widely used to precisely determine the mean number of photons in the input signal mode and compare it to other direct estimates based on power measurements and calibrated neutral density filters [25].

Heralded photon addition by stimulated PDC has been successfully used in a number of experiments and with a range of different input states. Among the main applications was the achievement of a tunable degree of nonclassicality (measurable by the negativity of the Wigner function or by other methods) for quantum light states [26,27,28].

### 2.2. Multimode Superposition of Heralded Single-Photon Additions

In general, the implementation of superpositions of heralded quantum operations relies on the indistinguishability of the herald photons coming from different processes, and it is obtained, for example, by mixing them on a beam-splitter before detection. Such approach was first used, in the case of photon addition and subtractions, to experimentally implement the commutator of bosonic creation and annihilation operators [9]. Properly mixing the herald photons from two photon subtraction stages placed, respectively, before and after a single-photon addition stage resulted in the indistinguishability and coherent superposition of the two inverse sequences of operators $\hat{a}{\hat{a}}^{†}$ and ${\hat{a}}^{†}\hat{a}$. However, in that case, the superposition of heralded operations took place on the same traveling mode of light.

If two or more conditional operations act on different field modes and their herald photons are mixed and made indistinguishable before detection, a more general multimode coherent superposition of quantum operations can be realized. An example of this approach in the case of a coherent superposition of photon subtractions was used to demonstrate the distillation of entanglement in a two-mode squeezed state [15].

**Figure 1:**
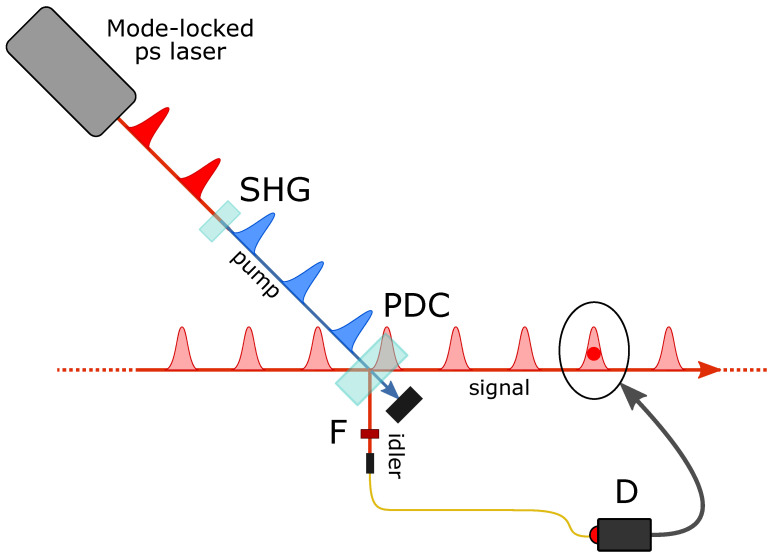
Experimental scheme for heralded single-photon addition. Symbols and abbreviations are defined in the text.

**Figure 2:**
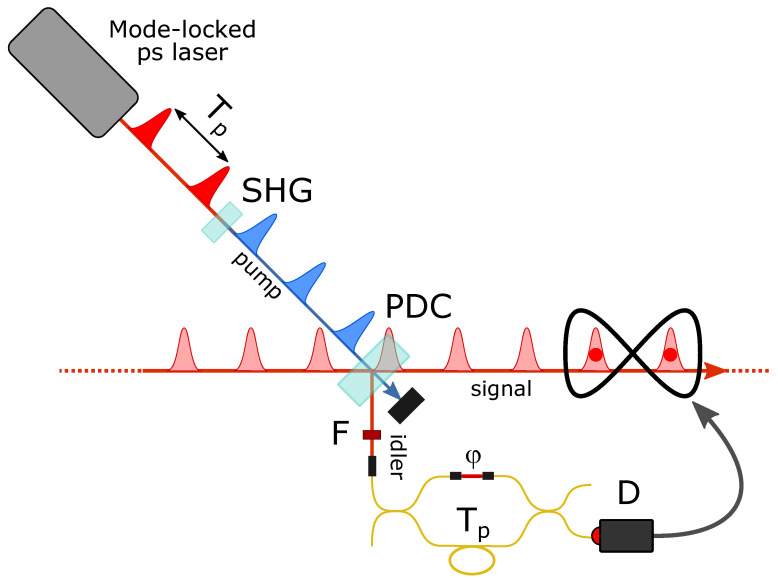
Experimental scheme for heralded multimode superposition of photon additions on consecutive temporal wavepacket modes by means of an unbalanced interferometer in the idler path.

The first superposition of multimode single-photon addition operations was realized by our group in 2006 [29]. Since its first demonstration, this experimental approach has been used in several different contexts and with different combinations of light states in the input modes. The particular scheme takes advantage of the pulsed operation of our main mode-locked laser source to remotely implement the coherent superposition of two photon addition operations on different traveling wavepacket temporal modes. Basically, the photon addition scheme described in Figure 1 is complemented by the introduction of an interferometer in the idler arm that allows the herald photons to travel paths of different length to reach the on–off photodetector, as shown in Figure 2. An idler photon detected by D may thus have been generated by either of two successive pump pulses, provided that the time delay between the short and long arms of the interferometer is chosen equal to the time separation $T_{p}$ between two successive pump pulses.

In general, both the reflectivity of the beam splitter and beam combiner in the interferometer and the fine relative phase offset φ from the precise pulse synchronization can be controlled and adjusted, so that a click in the idler detector may herald the success of the general superposition

(1) $$c_{1}{\hat{a}}_{1}^{†}+e^{i\varphi }c_{2}{\hat{a}}_{2}^{†},$$

where $c_{1}$ and $c_{2}$ are now real coefficients and the subscripts refer to the two successive temporal modes. A very interesting feature of the remote procedure for delocalized photon addition is that the losses experienced by the photons in the idler mode only contribute to a reduction in the overall success rate but do not influence the purity of the generated states. Therefore, one could simply generate unbalanced superpositions of photon additions by increasing the losses in one arm of the interferometer or, at least in principle, greatly increase the complexity of the herald optical circuit, for example by inserting additional arms of different delay in the interferometer in order to work on more temporal modes, without affecting the quality of the conditional superposition of operations. However, introducing losses or increasing the complexity of the heralding inevitably increases the measurement time and calls for a better and better stabilization of the optical setup in order to keep generating quantum light states of high purity.

Working with different temporal modes has important additional advantages over the possible use of distinct spatial ones. Besides the higher phase stability related to the sharing of the same single spatial mode by the different states of light, this approach also allows a much easier scalability because it allows one to increase the number of involved modes without a corresponding multiplication of PDC-based photon addition devices or detectors, as better shown in the next subsection.

The main difficulty in extending the setup shown in Figure 2 to more temporal modes would consist in the accurate phase control and stabilization of the different arms of the interferometer. In all cases studied so far, consisting of just two arms in a simple Michelson or Mach–Zehnder interferometer configuration, the state superposition phase φ is remotely controlled by varying the relative phase between the interferometer arms via a fine adjustment of an air-gap length. A feedback loop based on the interference of a counterpropagating pulse train injected in the unused interferometer output port provides phase stabilization.

### 2.3. Two-Mode Homodyne Detection and Tomographic State Reconstruction

The successful detection of a herald photon by the on–off photodetector placed at the output of the interferometer in the idler path conditionally implements a coherent superposition of photon additions on two different wavepacket temporal modes in the signal path. The resulting two-mode state, depending on the states originally populating the modes and on the particular superposition implemented, can be either directly analyzed by means of some characterization technique or, since it is produced in a heralded, non-destructive way, it can be used for further processing.

In order to study in detail the performance of the delocalized photon addition operation, we perform a quantum tomographic reconstruction of the output states analyzed by means of homodyne detection [30,31,32,33]. In a balanced homodyne detector, the state under investigation is mixed with a spatially and temporally mode-matched reference coherent field (the local oscillator, LO) at a 50% beam-splitter, and the intensities measured by two photodiodes placed at the output ports of the beam-splitter are subtracted. The difference photocurrent is proportional to the signal state quadrature $x_{\theta }$ at the phase θ of the LO field. In the case of a single temporal mode, a train of signal pulses is mixed with an attenuated train of coherent pulses coming from the laser and the outputs of the beamsplitter are detected by two fast proportional photodiodes. After amplification, the train of difference photocurrent pulses is analyzed by a digital oscilloscope triggered by herald click events. Quadrature data points are obtained by integrating the selected difference photocurrent signal over a time window of about 10 ns. The phase θ of the LO pulses relative to the signal mode ones is set by actuating a piezo-mounted (PZT) mirror in the LO path.

**Figure 3:**
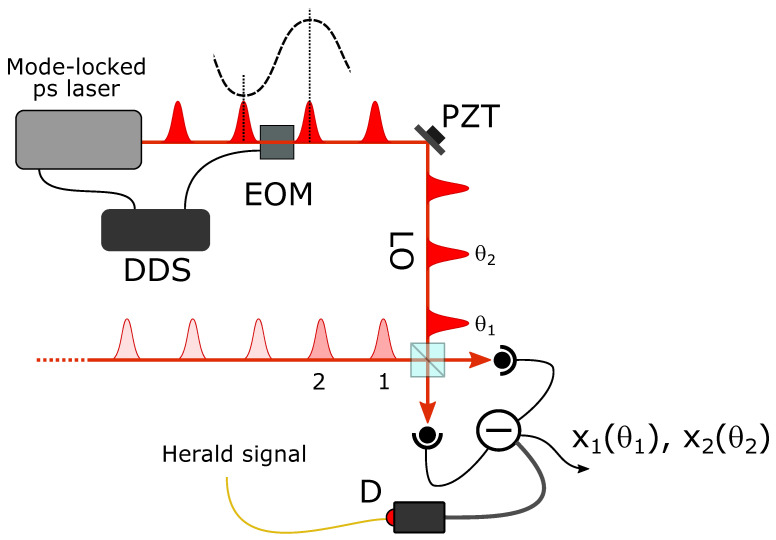
Scheme of the ultrafast modulation of the laser pulse train for the phase control of the local oscillator (LO) pulses in two-mode homodyne detection.

If the state occupies two (or more) temporal modes, a multimode extension of the above scheme is necessary [34]. In this case, a single herald click event triggers the acquisition of several consecutive difference photocurrent pulses and the corresponding quadrature data points are obtained by integrating over each mode of interest. Due to the relatively long temporal coherence of the mode-locked laser providing the LO pulse train, many successive LO pulses share essentially the same phase. However, for a full tomographic analysis and reconstruction of a multimode signal state, the phase $\theta_{i}$ of the LO pulses corresponding to the different *i* modes should, in principle, be individually controlled and adjusted. In the two-mode case, we independently changed the phases of the two LO pulses in the [0,π] interval by controlling their global phase via a piezo-mounted mirror and their relative phase by means of a fast electro-optic modulator (EOM), as shown in Figure 3. An ultraprecise timing system, based on a direct digital synthesizer (DDS), was used to generate the modulator driving signal, which is locked to the laser pulses, and synchronize the rest of the acquisition system.

After the acquisition of a sufficient number of quadrature measurements at different LO phases, the density matrix elements of the multimode states can be reconstructed by means of an iterative maximum likelihood algorithm [35,36]. A faithful representation of the states requires the reconstruction of a number of density matrix elements that grows extremely quickly with the number of photons in the input modes. For example, already considering two identical input coherent states with |α|≈7, corresponding to a mean photon number of $\bar{n}={\left|\alpha \right|}^{2}\approx 50$ photons per mode, at least $3\times 10^{7}$ density matrix elements need be calculated, and a brute force approach to full density matrix reconstruction has no hope to succeed with such a huge number of elements.

As shown below, a full independent control of all the LO phases or a full tomographic reconstruction of all the density matrix elements is not always strictly necessary. For particular states, or if one is interested in extracting only partial information about them, just the relative phase between the LO pulses needs be actively controlled, and the number of non-zero matrix elements to reconstruct can be drastically reduced.

### 2.4. Entanglement Measurements

In the following, we are interested in studying the degree of entanglement in the heralded states generated by the process of two-mode coherent photon addition. Nowadays, several techniques are available for this task. For example, if a description of the two-mode states via their density matrix $\hat{\rho }$ is available, one can measure the amount of entanglement by calculating the so-called negativity of the partial transpose (NPT) [37,38], which is proportional to the sum of the negative eigenvalues $\lambda_{i}^{-}$ of the partially-transposed density matrix ${\hat{\rho }}^{PT}$. The NPT is therefore defined as:

(2) $$NPT\left(\hat{\rho }\right)=-2\sum_{i}\lambda_{i}^{-},$$

where the factor 2 guarantees that $0\leq NPT(\hat{\rho })\leq 1$, being zero for separable states and 1 for maximally entangled ones. This approach allows one to quantify the amount of entanglement in the state [39]. However, many data are usually required to reconstruct the density matrix of multimode, multiphoton states, making the evaluation of $NPT(\hat{\rho })$ a demanding experimental task. Aiming at a less onerous entanglement detection, in the early 2000s, the concept of *entanglement witness* was introduced [40,41]. Formally, an entanglement witness is a Hermitian (thus measurable) operator ($\hat{W}$) for which stands:

(3) $$\begin{array}{cc} \; & Tr\left\{\hat{W}\hat{\rho }\right\}\geq 0\;\forall \hat{\rho }\in \left\{not\;entangled\;states\right\}\\ \; & Tr\left\{\hat{W}\hat{\rho }\right\}<0\;\forall \hat{\rho }\in \left\{entangled\;states\right\}. \end{array}$$

Even if they are not able to quantify entanglement, but just to detect its presence or not, entanglement witnesses require a much reduced amount of measurements to be evaluated. However, differently from the NPT criterion, which is a general condition to identify entanglement for a large class of quantum states [37,42], an entanglement witness can usually do so only for a small portion of them, making development hard from a theoretical point of view.

## 3. Results

In this section, we illustrate some representative examples of the application of two-mode coherent superposition of photon additions to various combinations of quantum light states populating the input temporal modes. We present the increasing number of interesting features and the corresponding growing degree of experimental complexity required as we move from simple vacuum inputs to more complex situations.

### 3.1. Delocalized Single Photon

**Figure 4:**
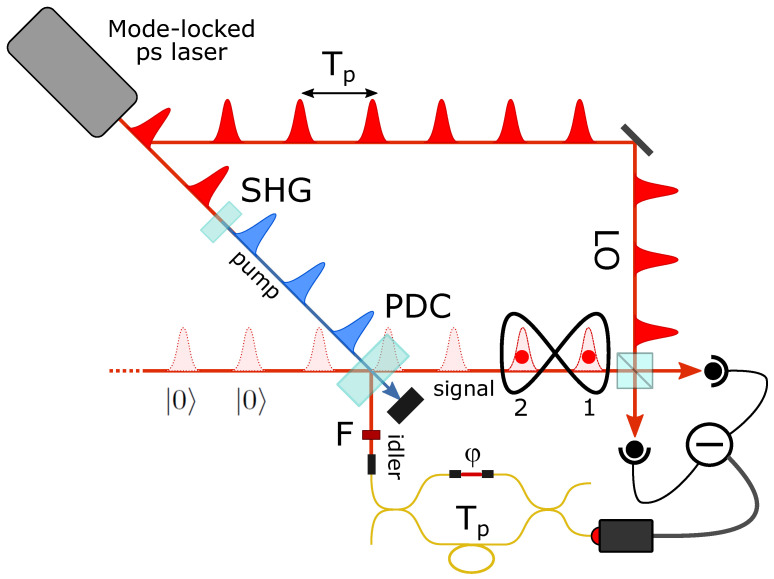
Scheme of the experiment for the heralded generation of a delocalized single photon.

The earliest and simplest example concerns the delocalized addition of a single photon to two empty distinct temporal modes [29], schematically illustrated in Figure 4. The application of the superposition of Equation (1) to the input quantum state ${|0\rangle }_{1}{|0\rangle }_{2}$ produces the single-photon mode-entangled state

(4) $$|\psi_{SP}\rangle_{12}=c_{1}{|1\rangle }_{1}{|0\rangle }_{2}+e^{i\varphi }c_{2}{|0\rangle }_{1}{|1\rangle }_{2},$$

which is normalized under the condition $c_{1}^{2}+c_{2}^{2}=1$. The reconstruction of its density matrix requires that, for each click in the herald on–off photodetector, one performs a homodyne measurement composed of a pair of quadrature values $x_{1}\left(\theta_{1}\right)$, $x_{2}\left(\theta_{2}\right)$ with the phases $\theta_{1}$ and $\theta_{2}$ defined by the phases of local oscillator pulses at temporal modes 1 and 2. In principle, one should acquire quadrature measurements while performing a full scan of the two phases $\theta_{1}$ and $\theta_{2}$ independently. However, one can easily demonstrate that, as long as one of the states in the two input modes is a Fock state, the two-mode quadrature probability distributions only depend on the LO phase $\theta_{2}$ and the sum of the LO phase in the first mode $\theta_{1}$ and the superposition phase φ, whose roles are thus interchangeable. Therefore, for the class of states including that of Equation (4), it is possible to perform quantum tomography by using a common local oscillator phase for the two modes ($\theta =\theta_{1}=\theta_{2}$) and varying the state superposition phase φ, instead of keeping the state fixed and scanning the two LO phases during the homodyne acquisition (see the Supplementary Materials of [43]). This property makes the experimental setup particularly simple, as any two consecutive equal-phase pulses coming from the mode-locked train can be directly used as LO pulses while remotely varying the phase φ by means of the interferometer in the idler channel. Moreover, since the simple one-photon state of Equation (4) is invariant with respect to global phase shifts, one does not even need to control and stabilize the global LO phase θ with the piezo-mounted mirror.

Upon detection of each idler photon, the homodyne signals of the two corresponding consecutive signal temporal modes (plus a third one containing just vacuum and used for calibration) are acquired by the digital oscilloscope, and quadrature values for each mode are extracted. A total of $10^{6}$ quadrature measurements for each mode, equally distributed over the range [0,π] of φ, allows the reconstruction of the two-mode density matrix of the quantum state truncated to a maximum number of Fock state contributions of n=2, for a total of $3^{4}=81$ density matrix elements. With a full reconstruction of the density matrix from homodyne quadrature measurements, the entanglement of this kind of state was clearly demonstrated [29,44].

**Figure 5:**
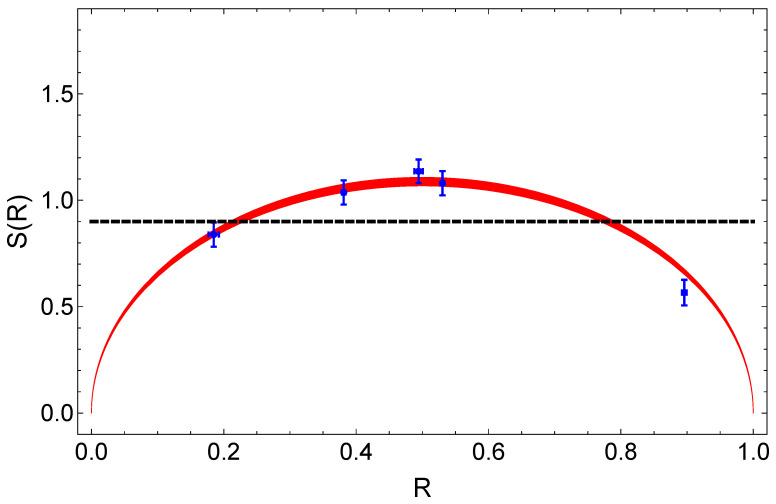
Experimental values (blue dots) of the entanglement witness S(R) in Equation (6), as a function of the superposition weight *R*, measured on the states of Equation (4). The black dashed line is the theoretical threshold above which S(R) detects entanglement. Experimental values larger than this threshold certify the presence of entanglement in the state, whereas values below it cannot reveal any information about this property. The red area represents the theoretical behavior of the witness calculated on the states of Equation (4), considering fluctuations in the detection efficiency during the measurements.

Even if the experimental and computational efforts required for the reconstruction of such a moderate-size density matrix are not too demanding, interesting witnesses that are able to efficiently check the presence of entanglement in this kind of single-photon path-entangled states have been developed. As an example, we show in Figure 5 the results that we obtained by evaluating the witness proposed by Morin et al. [45] on states of the form of Equation (4), generated while scanning the weights of the superposition. With this approach, the presence of entanglement can be witnessed by measuring the quadratures of the two modes for just a small set of relative phases between the two LO pulses ($\theta_{1}-\theta_{2}=\left\{-\pi /4,\pi /4,3\pi /4\right\}$). Again, this relative LO phase can be equivalently switched with the superposition phase set by the interferometer ($\theta_{1}-\theta_{2}↔\varphi$). The results shown in Figure 5 are obtained following this scheme. For each value of φ, one should evaluate the probability of measuring, at each click of the heralding detector, quadrature values with equal or opposite sign in the two modes. This way, it is possible to evaluate the parameter

(5) $$E_{\varphi }\left(R\right)=P\left(a=b|x_{1},x_{2}\right)-P\left(a\neq b|x_{1},x_{2}\right),$$

where *a* and *b* represent the signs of the measured $x_{1}$ and $x_{2}$, respectively. *R* corresponds to $c_{1}$ of Equation (4) and can thus be varied, adjusting the superposition weights, by modifying the reflectivity of the beam-splitters of the interferometer in the idler path.

The witness can be constructed by combining the values of Equation (5) measured for all the values of φ as:

(6) $$S\left(R\right)=E_{\varphi =-\frac{\pi }{4}}\left(R\right)+2E_{\varphi =\frac{\pi }{4}}\left(R\right)-E_{\varphi =\frac{3\pi }{4}}\left(R\right).$$

The maximum value of Equation (6) that can be obtained measuring separable states is $2\sqrt{2}/\pi ∼0.9$. Values above this threshold witness entanglement in the measured state. Intuitively, the detection efficiency (η) of the setup affects the performances of this witness. The theoretical behavior of S(R), taking into account the experimental imperfections, is represented by the red region in Figure 5. This region is obtained considering a mean value of η of 60.4%, with an overall variation of 3.5% during the entire measurement period. The very good agreement between the experimental data and the theoretical model confirms the effectiveness of the witness [45]. Remarkably, our measurements are able to certify the presence of entanglement for the class of states described by Equation (4) in a very wide range of superposition weights (0.22±0.06)≤R≤(0.78±0.07).

### 3.2. Hybrid Entanglement

A big step forward in the complexity of the experimental setup for superposed single-photon addition and the analysis procedure is constituted by the generation of the so-called hybrid entangled states [43,46]. These states present entanglement between two distinct modes where the light states are best described in the discrete- (DV) or continuous-variable (CV) type of encoding, respectively. Single photons are typical examples of DV states, and typical DV qubits can be made of superpositions of the presence and absence of a single photon in a particular field mode. Conversely, coherent states of light are typical examples of CV states, and CV qubits can be made of the superposition of two coherent states of different complex amplitude.

An hybrid DV-CV entangled state may thus have the form

(7) $$|\psi_{H}\rangle_{12}=\frac{1}{\sqrt{2}}{(|1\rangle }_{1}{|\alpha \rangle }_{2}+e^{i\varphi }{|0\rangle }_{1}|\alpha^{\prime }\rangle_{2})$$

and it can be approximately produced by a coherent superposition of single-photon addition operations onto two distinct temporal modes containing vacuum and a coherent state, ${|0\rangle }_{1}{|\alpha \rangle }_{2}$ [47]. In fact, it is easy to observe that a single-photon-added coherent state [1,48], the result of the application of the photon creation operator onto a coherent state |α〉, can well approximate another coherent state of slightly larger amplitude $|\alpha^{\prime }\rangle$, whenever |α| is sufficiently large.

**Figure 6:**
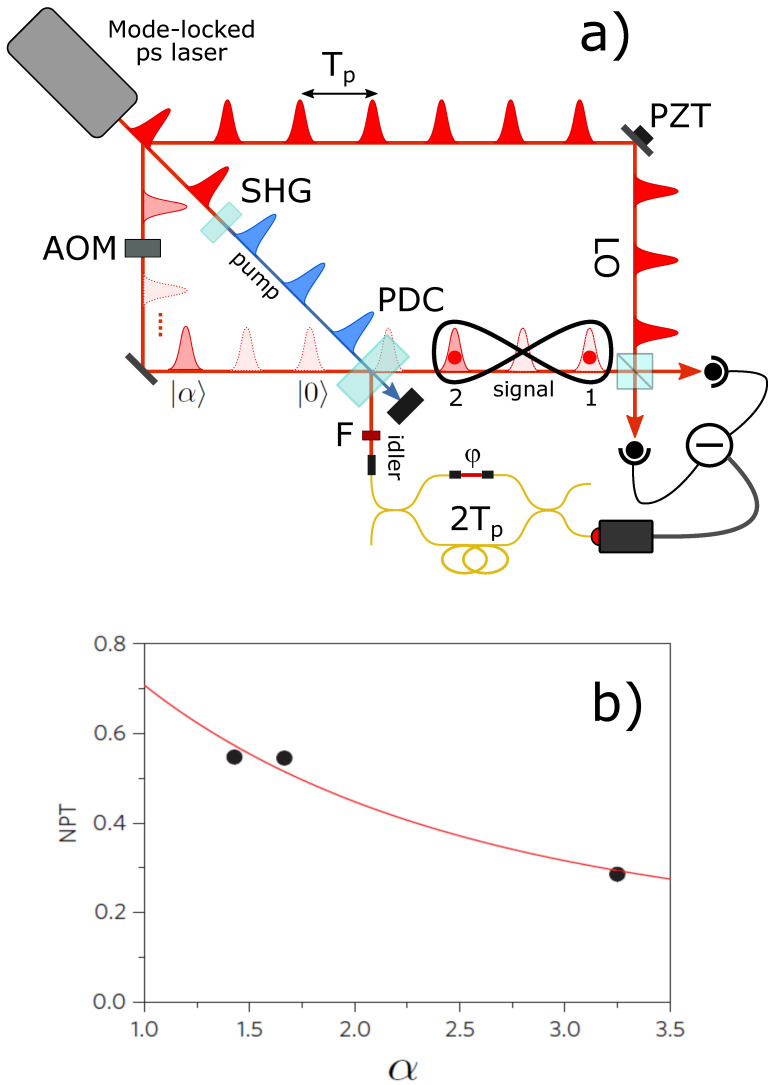
(**a**) Experimental scheme for hybrid entangled state generation. (**b**) Measured values and theoretical behavior of the NPT as a function of the amplitude α of the input coherent state.

However, in this case, the simple balanced superposition of photon additions described by Equation (1) does not directly produce the desired balanced entangled state of Equation (7). In fact, since the photon addition in the second temporal mode is now a stimulated process, it is more likely to occur than the spontaneous one in the first by a factor $1+{\left|\alpha \right|}^{2}$. Therefore, the amplitudes of the two photon addition operators in the superposition of Equation (1) have to be properly adjusted as

(8) $${\hat{a}}_{1}^{†}+\frac{e^{i\varphi }}{\sqrt{1+{\left|\alpha \right|}^{2}}}{\hat{a}}_{2}^{†}$$

by varying the reflectivity of the first interferometer beam-splitter according to the amplitude of the input coherent state. In the experiment, schematically illustrated in Figure 6a [49], this is achieved by adjusting a variable-ratio fiber coupler at the input of the interferometer so as to equalize the idler count rates from each of the two interferometer arms.

In order to prepare the required input state, that is one composed of vacuum in the first temporal mode and a coherent state |α〉 in the second, an acousto-optic modulator (AOM) is inserted in the path of an attenuated portion of the laser pulse train to work as a pulse-picker. In principle, one should operate the AOM at a rate such that it transmits every other pulse of the laser train. However, bandwidth limitations in the AOM driver and trigger electronics led us to use a lower rate of about 10 MHz. The AOM thus transmits one in every eight pulses of the train and reduces the acquisition rate of the experiment at least by a factor of 8.

After injection of the seed pulse train in the parametric crystal along the signal spatial mode, an idler photon detection by the on–off photodetector at the output of the interferometer heralds the delocalized photon addition of Equation (8) on the two signal temporal modes labeled as 1 and 2. Although these two modes could be separated by exactly the interpulse delay $T_{p}\approx 12$ ns given by the laser repetition rate, as in the previous experiment, we chose to use $2T_{p}$ instead, thus encoding the analyzed temporal modes in non-consecutive pulses, to improve the discrimination of the modes during homodyne detection. Homodyne quadrature measurements involving both the temporal modes of interest are synchronized to the periodic 10 MHz signal used to drive the AOM (thus guaranteeing the presence of the input signal state ${|0\rangle }_{1}{|\alpha \rangle }_{2}$) and triggered by the detection of an idler photon after the interferometer.

Since, in this case as well, one of the two input modes contains a Fock state, one can take advantage of the interchangeability between the relative phases of the LO pulses and the phase of the superposition to simplify the setup by scanning the latter (we use nine values in the [0,π] interval) and using same-phase LO pulses for the homodyne analysis. However, differently from the delocalized single-photon case, here, the common phase of the LO pulses with respect to the input coherent state also has to be controlled and properly scanned. This is done by acting on a piezo-mounted steering mirror to lock the mean value of the homodyne photocurrent output at different positions of the interference fringes between the LO pulses and the seed coherent state pulses.

About $6\times 10^{5}$ quadrature pairs are acquired to perform a full tomography of the state using a two-mode maximum-likelihood-based algorithm [35,36]. The dimensions of the reconstructed density matrices in the Fock basis are adjusted to the size of the investigated states [50]. Therefore, 3 terms in the Fock expansion are used here for the first mode (ideally containing just vacuum and single-photon components), whereas up to 25 are necessary for the second mode, containing the coherent and the photon-added coherent states. The experimental NPT values extracted from the reconstructed density matrices corrected for detection efficiency are used to quantify the entanglement of the hybrid state and are reported in Figure 6b. The state presents a degree of entanglement that decays with the amplitude of the coherent component α, in full agreement with the expected NPT behavior of the state resulting from the application of the superposition of Equation (8).

### 3.3. Entanglement of Macroscopic Coherent States

The effect of a coherent superposition of photon addition operations is finally experimentally evaluated in the case of two input modes containing identical coherent states |α〉 [51]. The state produced by the balanced superposition of Equation (1) on the input state ${|\alpha \rangle }_{1}{|\alpha \rangle }_{2}$ can be written as:

(9) $$\begin{array}{ccc} |\psi_{\varphi }{\left(\alpha \right)\rangle }_{12} & = & [{\hat{D}}_{1}\left(\alpha \right){\hat{D}}_{2}\left(\alpha \right)\left({|1\rangle }_{1}{|0\rangle }_{2}+e^{i\varphi }{|0\rangle }_{1}{|1\rangle }_{2}\right)\\ & + & \alpha^{*}\left(1+e^{i\varphi }\right){|\alpha \rangle }_{1}{|\alpha \rangle }_{2}]/\sqrt{N} \end{array}$$

with the normalization factor $N=2[1+|\alpha {|}^{2}\left(1+\cos\varphi \right)]$ and the phase-space displacement operator defined as $\hat{D}\left(\alpha \right)=e^{\alpha {\hat{a}}^{†}-\alpha^{*}\hat{a}}$. The output state is made of an entangled and a separable part, whose relative weights depend on the superposition phase φ and the amplitude α of the coherent states. When φ=0, the entanglement of the state decreases for increasing α, due to the growing contribution of the separable fraction. When the other extreme condition of φ=π is reached, the separable part disappears and the resulting state reduces to a displaced delocalized single photon. Since displacing a state in phase space does not change its entanglement, one can preserve it at its constant maximum value independently of the amplitude of the input coherent states and even between two modes initially containing large mean photon numbers $\bar{n}={\left|\alpha \right|}^{2}$. Such a state, characterized by a degree of entanglement independent of the size of the entangled partners and surprisingly robust against losses [52], is of very high interest to investigate the resilience and detectability of entanglement for states of growing macroscopicity as well as for fundamental investigations concerning the very definition of macroscopic quantumness and entanglement [53,54,55]. Related experiments involving micro–macro [56,57] and macro–macro [58] entanglement have recently explored these issues, with different approaches.

**Figure 7:**
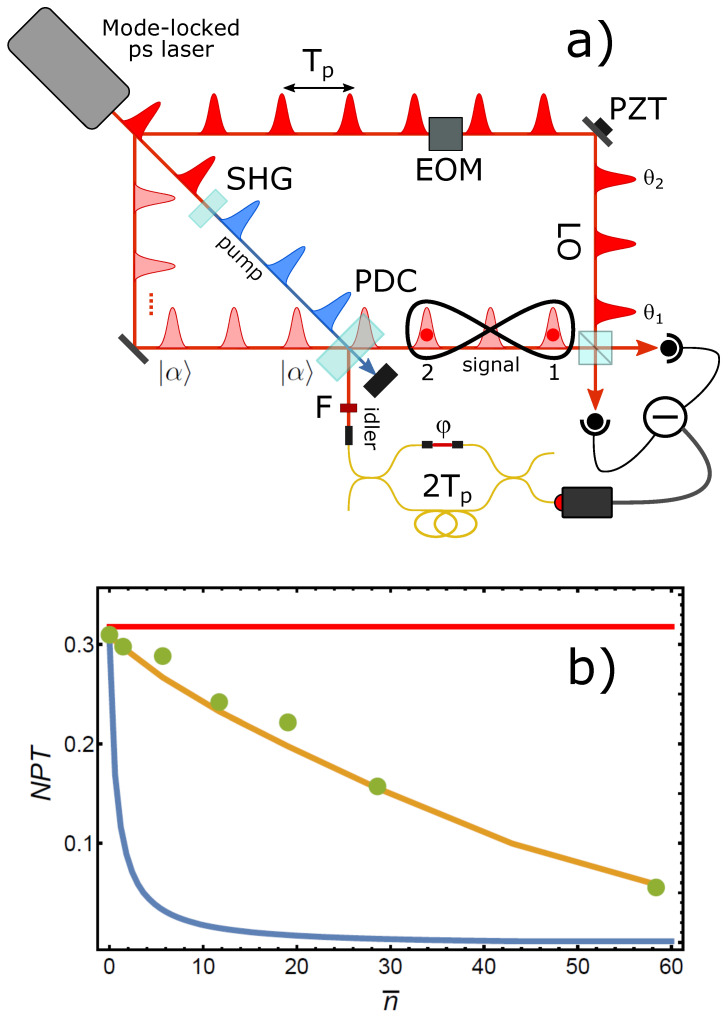
(**a**) Experimental scheme for the generation of macro-macro entanglement. (**b**) Experimental NPT (green dots) of the generated states as a function of the mean number of photons $\bar{n}$ in the input coherent states. Red and blue solid curves are the ideal NPT for φ=π and φ=0, respectively. The orange curve is calculated for φ=π when all the experimental imperfections are considered.

The setup to produce and analyze such states has to be further modified with respect to the previous experiments (see Figure 7a). Since in this case the input signal temporal modes have to be populated by identical coherent states, the previously used AOM is no longer needed, and an entire attenuated version of the laser pulse train is injected in the parametric crystal along the signal spatial mode. Also in this case, to reduce any contamination between different temporal modes during homodyne detection, we set the delay between the two arms of the interferometer to $2T_{p}$, thus encoding the analyzed temporal modes 1 and 2 in non-consecutive pulses. Being only interested in the superposition of photon addition operations with a well-defined phase of φ=π, the interferometer is locked at this value, which corresponds to a minimum in the count rate of the on–off idler photodetector. Since neither of the input modes contains a Fock state, a complete scan of both LO pulse phases is now required, and it is implemented by actuating both the piezo-mounted mirror and the ultrafast phase modulator synchronized to the laser repetition rate.

As we are interested in studying the entanglement produced between two modes that may contain more than just a few, and possibly many, photons, a full reconstruction of the state density matrix is computationally prohibitive, and we therefore have to adopt a different strategy here. From Equation (9), it is evident that the entanglement features of the state with φ=π fully derive from those of a displaced delocalized single photon and are thus entirely expressed by the correlated fluctuations of the quadrature measurements of the two modes. Therefore, one can just use such fluctuations around their common mean values for tomographic reconstruction of the two-mode density matrix in a reduced Fock subspace of zero, one and two photons. About 50,000 quadrature values are acquired for nine different relative LO phases with the global LO phase locked to zero, and the means of the measured quadrature distributions are subtracted before reconstruction. This procedure allows one to keep the dimensions of the reconstructed Fock space fixed regardless of the state macroscopicity, thus allowing the measurement of entanglement for very large states. Applying this procedure allowed us to measure the NPT values represented by the green dots in Figure 7b. The red (blue) solid curve in the figure represents the behavior of the NPT for the ideal states of Equation (9) for the case of φ=π(0); the orange solid curve is the calculated NPT for φ=π when all experimental imperfections are accounted for [51]. Interestingly, although the experimental NPT shows a decay due to a degradation in the state preparation procedure for growing $\bar{n}$ [51], the analyzed states nonetheless preserve a relatively large degree of entanglement even for macroscopic mean photon numbers (up to $\bar{n}\approx 60$) in each mode.

Differently from other recent experimental approaches [56,57,58], in our experiment, the two-mode entangled state is always fully detected by the homodyne detector in its complete macroscopic form, without resorting to displacing it back to the limited Fock space spanned by the vacuum and single-photon components before measurement. Therefore, in our case, one can use the full quadrature measurements (comprising both the quadrature mean values and their fluctuations) in the two modes for extracting other important parameters of the state, such as some entanglement witness or some particular joint statistical properties.

**Figure 8:**
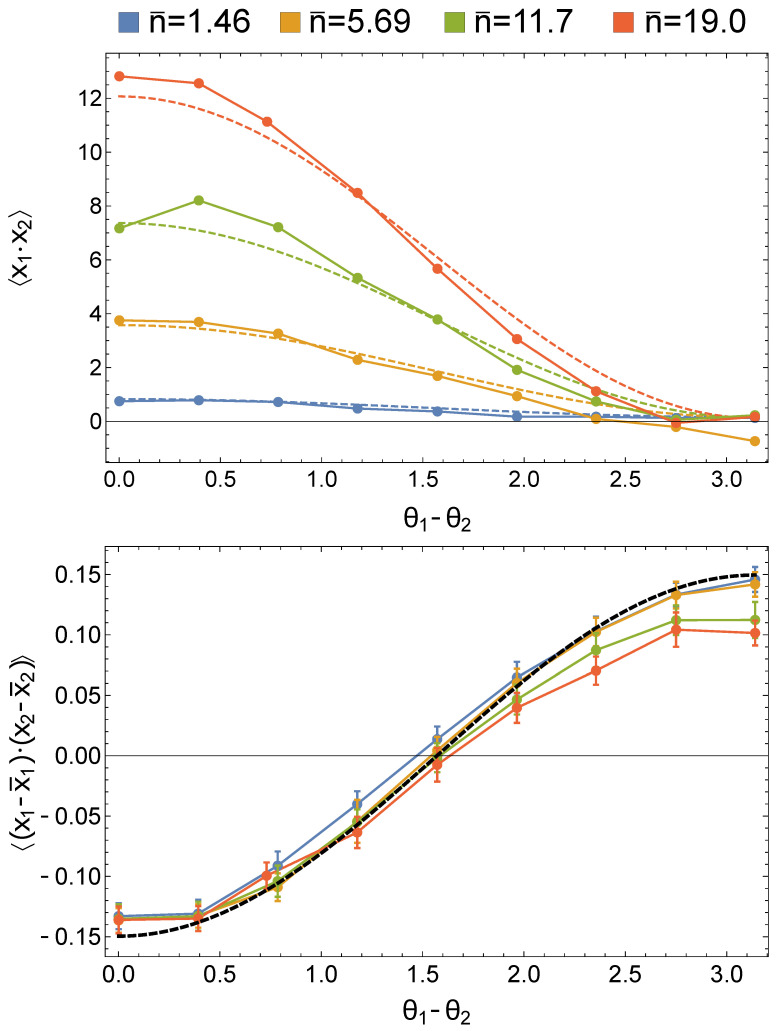
(**Top**) Raw correlations between the quadratures in the two modes as acquired from homodyne detection. Dots represent experimental points for different values of $\bar{n}$, while dashed lines are the theoretical predictions considering the measured experimental inefficiencies. (**Bottom**) Quadrature correlations for different values of $\bar{n}$ after removal of the mean field from the measured quadrature distributions. Dots are the experimental points, while the black dashed line is the theoretical prediction for a balanced single-photon path-entangled state, with a detection efficiency of $\eta_{p}=0.92$.

As an example, it is possible to directly study the product of the quadratures measured by homodyne detection for the two modes as a function of the relative phases of the local oscillator pulses. Figure 8 (top) reports the behavior of such a product of quadratures for different values of the mean photon number of the coherent states seeding the PDC crystal.

As expected from the theoretical dashed lines also reported in the figure, the quadratures in the two modes are positively correlated, with a strength increasing with $\bar{n}$, for $\theta_{1}=\theta_{2}$, while such a positive correlation reaches its minimum value when $\theta_{1}-\theta_{2}=\pi$. On the contrary, the removal of the mean field contribution from the quadrature data before calculating their product has the result of going back to the simple situation of a path-entangled single photon. In this case, a clear anti-correlation in the two modes is present for $\theta_{1}=\theta_{2}$, whereas the quadratures are uncorrelated or positively correlated for $\theta_{1}-\theta_{2}=\pi /2$ or $\theta_{1}-\theta_{2}=\pi$, respectively.

### 3.4. Discorrelation

Discorrelation [59] is another joint statistical property of multimode quantum light states, which involves photon numbers instead of quadratures. In discorrelated states, the number of photons in each mode can take any value individually, but two modes together never exhibit the same. For a two-mode state, it manifests itself in null diagonal elements of the joint photon number probability distribution $P_{n_{1},n_{2}}$, where $n_{1}$ and $n_{2}$ are the numbers of photons in the two modes, i.e., $P_{n,n}=0$, while the marginal distributions $P_{n_{1}}=\sum_{n_{2}=0}^{\infty }P_{n_{1},n_{2}}\neq 0$, for any value of $n_{1}$. The discorrelation property is, in some sense, the opposite of the perfect photon number correlation existing in ideal two-mode squeezed vacuum states, where two perfect detectors always measure the same number of photons in the idler and signal modes. Therefore, opposite to quantum key distribution schemes, where common random keys need to be shared [60,61], discorrelation can be used to distribute unique randomness between parties. In particular, it could be useful in so-called ‘mental poker’ problems, which are concerned with the fair dealing of cards between distant players without a trusted third party [62,63].

It is easy to see that the same state analyzed above, resulting from the balanced odd superposition of photon additions on two modes containing identical coherent states and described by Equation (9) with φ=π, presents perfect discorrelation. The most direct way to study its discorrelation properties would imply a joint photon-number-resolving measurement [64] on the two consecutive wavepacket modes. However, one may also follow a different, indirect, strategy involving the reconstruction of the density matrix fully representing the optical two-mode state. Since $P_{n_{1},n_{2}}$ is invariant with respect to the LO global phase θ, we performed a tomographic reconstruction by averaging θ by means of the PZT and acquiring about 50,000 quadratures for 9 equidistant LO relative phase values in the interval [0,π] by means of the EOM. This reduces the number of non-zero density matrix elements and makes the maximum likelihood algorithm computationally accessible for the full reconstruction of states with $\bar{n}≲5$ in each mode.

**Figure 9:**
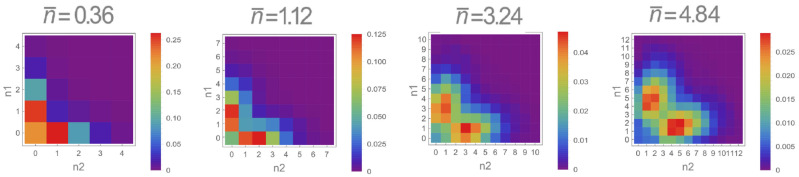
Raw joint photon number probability distributions as a function of the mean number of photons $\bar{n}$ of the input coherent states, clearly showing the effect of discorrelation.

The joint photon number distributions $P_{n_{1},n_{2}}^{e}$ obtained from experimentally reconstructed density matrices without correction for detection efficiency are shown in Figure 9 for different amplitudes of the input coherent states. Although the experimental distributions do not exhibit a perfectly null diagonal due to experimental imperfections (limited detection efficiency and imperfect delocalized photon addition operation), they nevertheless present an evident decrease in the magnitude of the diagonal elements, a clear signature of discorrelation.

## 4. Discussion and Conclusions

We have shown how the simple process of coherently adding a single photon onto different field modes can produce quantum states of light characterized by interesting and unique properties. Thanks to the many parameters that can be controlled in the process (from the weights and relative phases of the superposition of photon creation operations to the states initially populating the modes), a huge variety of quantum states can be generated, and their properties, such as entanglement and discorrelation, may be arbitrarily tuned in a controllable way. Although several important results have already been achieved and are briefly described here, much remains to be explored. For example, different kinds of states, other than vacuum and coherent, could be considered at the input: from purely classical ones, such as thermal states, to highly nonclassical ones, such as squeezed vacuum and squeezed coherent states. Besides their fundamental interest, such investigations may have important consequences for possible applications in the context of quantum technologies. For example, as quickly shown above, the discorrelation feature could find use in particular quantum communication schemes, while the high sensitivity of the entanglement between macroscopic light states on the phase of the coherent superposition might be applied for quantum sensing purposes [65]. More generally, the results briefly illustrated in this review and future ones along these lines might open new avenues for technologies at the intersection of quantum-enhanced sensing and quantum information processing and communication.

## Data Availability

The datasets generated and analyzed during the current study are available from the corresponding authors upon reasonable request.
